# An RNAi screen for secreted factors and cell-surface players in coordinating neuron and glia development in Drosophila

**DOI:** 10.1186/s13041-019-0541-5

**Published:** 2020-01-03

**Authors:** Zhengya Liu, Yixu Chen, Yong Rao

**Affiliations:** 1 Centre for Research in Neuroscience, McGill University Health Centre, Room L7-136, 1650 Cedar Avenue, Montreal, Quebec H3G 1A4 Canada; 2 Integrated Program in Neuroscience, McGill University Health Centre, 1650 Cedar Avenue, Montreal, Quebec H3G 1A4 Canada; 3 Department of Neurology and Neurosurgery, McGill University Health Centre, 1650 Cedar Avenue, Montreal, Quebec H3G 1A4 Canada

**Keywords:** Photoreceptor, Glia, Coordinated development, Amalgam, Neuroglian, RNAi screen, Drosophila

## Abstract

The establishment of the functional nervous system requires coordinated development of neurons and glia in the embryo. Our understanding of underlying molecular and cellular mechanisms, however, remains limited. The developing *Drosophila* visual system is an excellent model for understanding the developmental control of the nervous system. By performing a systematic transgenic RNAi screen, we investigated the requirements of secreted proteins and cell-surface receptors for the development of photoreceptor neurons (R cells) and wrapping glia (WG) in the *Drosophila* visual system. From the screen, we identified seven genes whose knockdown disrupted the development of R cells and/or WG, including *amalgam (ama)*, *domeless (dome)*, *epidermal growth factor receptor (EGFR), kuzbanian (kuz)*, *N-Cadherin (CadN)*, *neuroglian (nrg),* and *shotgun (shg)*. Cell-type-specific analysis revealed that *ama* is required in the developing eye disc for promoting cell proliferation and differentiation, which is essential for the migration of glia in the optic stalk. Our results also suggest that *nrg* functions in both eye disc and WG for coordinating R-cell and WG development.

## Introduction

Building brain architecture requires the coordinated development of neurons and glia. In mammals, it is shown that neuronal-derived signals promote the proliferation and differentiation of glia such as astrocytes and oligodendrocytes [1, 2]. Accumulated evidence also supports that glia would actively regulate neuronal differentiation and function [3–5]. A comprehensive understanding of coordinated neuronal and glial development requires the identification and characterization of important players involved.

The *Drosophila* visual system is an excellent model for understanding the control of coordinated neuron and glia development. Photoreceptor neurons (R cells) and wrapping glia (WG) originate from different tissue compartments. R cells are born in the eye-imaginal disc, an epithelial monolayer covered by the peripodial membrane, at the third-instar larval stage [6]. In the developing eye disc, precursor cells located posterior to the morphogenetic furrow undergo differentiation, and give rise to eight different R cells: R8 differentiates first, followed by R2/5, R3/4, R1/6, and R7. R cells project axons from the eye disc through the optic stalk into the developing optic lobe. Sub-retinal glia originate in the optic stalk. At the third-instar larval stage, perineurial glia (PG) migrate from the optic stalk into the sub-retinal region where they differentiate into WG after contacting nascent R-cell axons [7].

Recent studies identify several neuron-derived factors that coordinate the development of R cells and WG [8, 9]. It is shown that the neuron-derived FGF8-like ligand Thisbe promotes the differentiation of PG into WG, which migrate along the surface of R-cell axons and subsequently insulate R-cell axons [8]. Our previous studies reveal that the immunoglobulin (Ig) superfamily transmembrane protein Turtle (Tutl) expressed on R-cell axons binds to the WG-specific cell-surface receptor Borderless (Bdl) to promote WG extension and axonal ensheathment [9, 10]. While it is reported that WG also plays an active role in regulating the topographic projection of R-cell axons in the optic lobe [11], the underlying mechanisms remain unclear.

To identify additional cell-surface players that are involved in regulating the coordinated development of R cells in the eye disc and WG in the sub-retinal region, we set out to perform a transgenic RNAi screen targeting 177 secreted proteins and cell-surface receptors (Additional file 1: Table S1). From the initial screen, we identified thirteen RNAi lines that disrupted the development of R cells and/or WG. By testing additional RNAi lines, we confirmed seven genes, including *amalgam (ama)*, *domeless (dome)*, *epidermal growth factor receptor (EGFR), kuzbanian (kuz)*, *N-Cadherin (CadN), neuroglian (nrg),* and *shotgun (shg)*. Cell-type-specific knockdown experiments show that while *nrg* acts in both eye disc and WG, the remaining six genes are only required in the developing eye disc for R-cell and WG development.

## Results

### Transgenic RNAi screen for abnormal development of R cells and WG in the developing *Drosophila* visual system

To identify novel cell-surface players in coordinating the development of R cells and WG, we performed a systematic transgenic RNAi screen targeting 177 genes that encode for secreted proteins and cell-surface receptors (Additional file 1: Table S1).

**Fig. 1:**
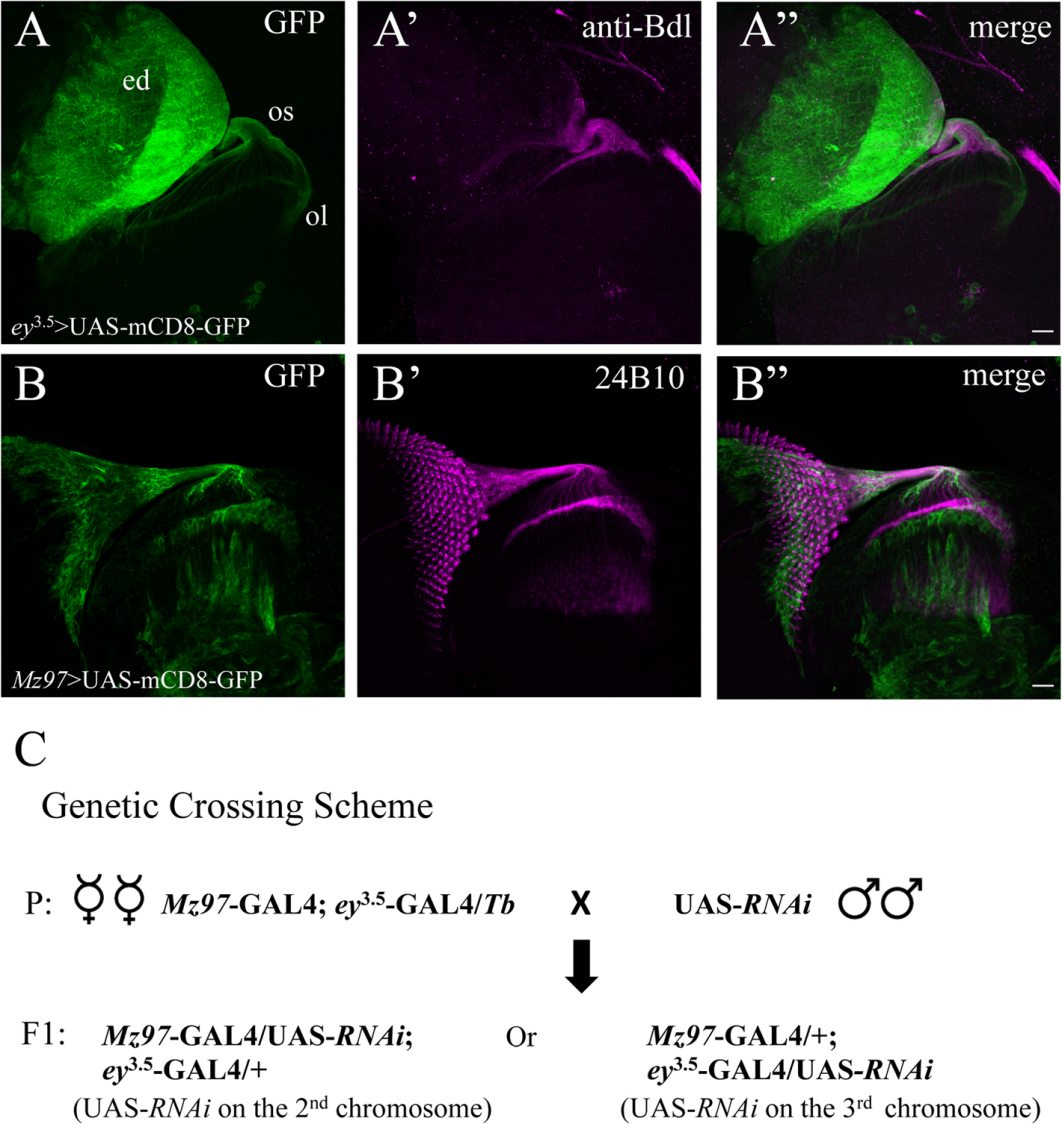
A transgenic RNAi screen targeting secreted proteins and cell-surface receptors in the developing visual system. A-A” and B-B″, 3rd-instar larval eye-brain complexes were double-stained to visualize the expression pattern of UAS-mCD8-GFP under control of the eye-specific *ey*^3.5^-GAL4 or WG-specific *Mz97*-GAL4 drivers. A-A”, 3rd-instar larval eye-brain complexes carrying UAS-mCD8-GFP under control of *ey*^3.5^-GAL4 were labeled with GFP fluorescence (green) and anti-Bdl staining (magenta). *ey*^3.5^-GAL4 drove the expression of target genes in all cell types in the epithelial monolayer of eye disc (ed), but not in WG. B-B″, 3rd-instar larval eye-brain complexes carrying UAS-mCD8-GFP under control of *Mz97*-GAL4 were labeled with GFP fluorescence (green) and MAb24B10 staining (magenta). *Mz97-*GAL4 drove gene expression in WG as well as some other glial types in the optic lobe, but not in the epithelial monolayer of eye disc. C, A scheme of the RNAi screen. UAS-*RNAi* transgenes were simultaneously expressed in the eye disc and sub-retinal WG under control of both *ey*^3.5^-GAL4 and *Mz97*-GAL4. Abbreviations: ed., eye disc; ol, optic lobe; os, optic stalk. Scale bar: 20 μm

**Fig. 2:**
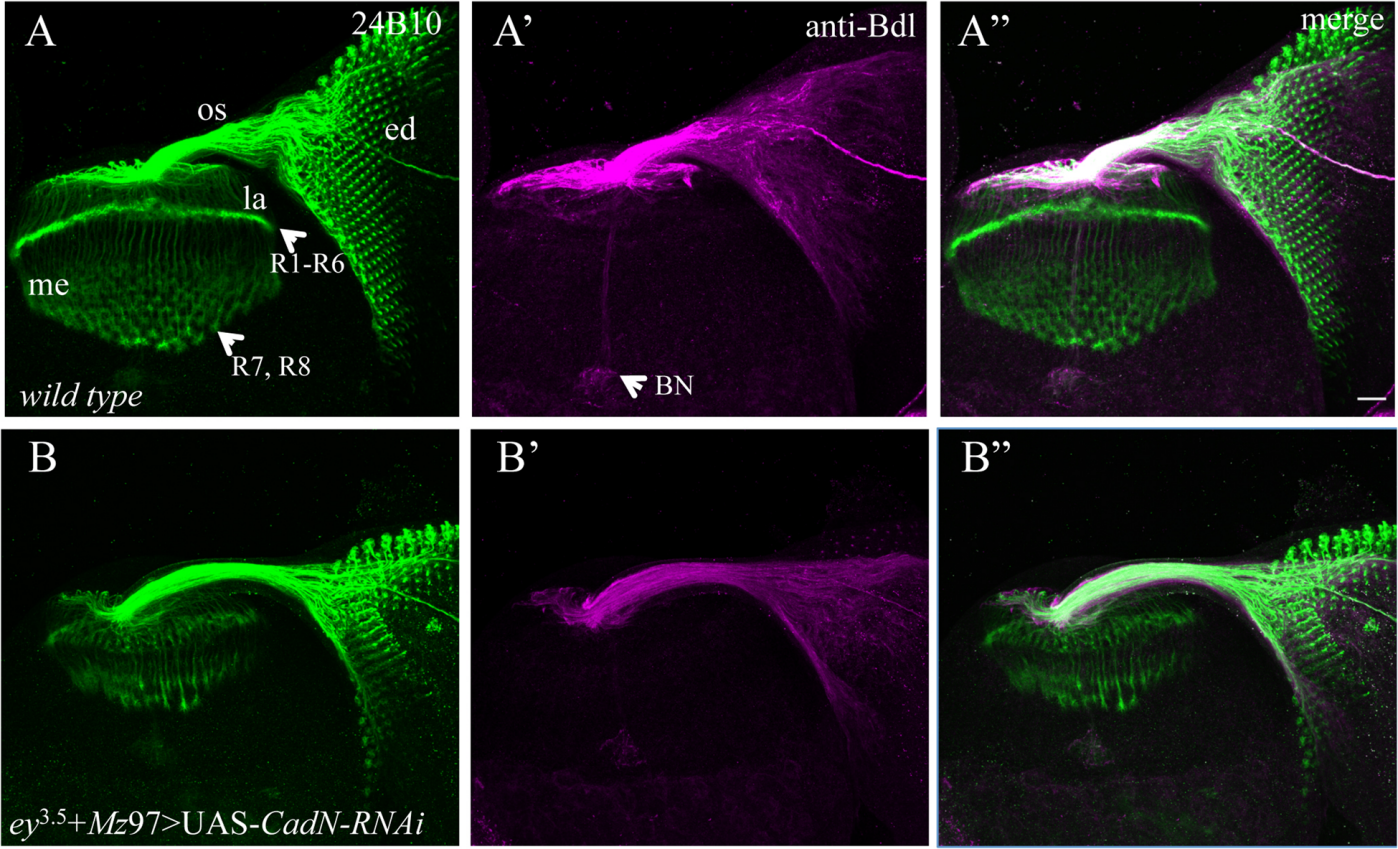
Knockdown affected R-cell development only. Candidate genes were knocked down simultaneously in both R cells and WG by expressing the UAS-*RNAi* transgene under control of *ey*^*3.5*^-GAL4 and *Mz97*-GAL4. 3rd-instar larval eye-brain complexes were double-stained with MAb24B10 (green) and anti-Bdl (magenta). A-A”, the projection pattern of R-cell axons and WG processes in wild type (100%, *n* = 15). A, the section was stained with MAb24B10 only. R-cell axons migrate towards the posterior end of the eye disc (ed) and subsequently enter the optic stalk (os). After exiting the optic stalk, R1-R6 axons terminate at the lamina (la), where their expanded growth cones form a dense and continuous layer. R7 and R8 axons pass through the lamina and form a regular array of terminals within the deeper medulla layer (me). A’, the same section was stained with anti-Bdl to visualize WG. WG processes follow R-cell axons from the eye disc through the optic stalk into the lamina. BN, Bolwig’s Nerve. A”, the section was visualized with both MAb24B10 and anti-Bdl staining. B-B″, a third-instar eye-brain complex in which *CadN* was simultaneously knocked down in both eye disc and WG. *CadN* knockdown disrupted the termination pattern and the morphology of R-cell axons (B and B″), but did not affect WG development (B’ and B″). Scale bar: 20 μm

To simultaneously knock down a candidate gene in both R cells and WG, the UAS-*RNAi* transgene was expressed in R cells and WG under control of *ey*^*3.5*^-GAL4 and *Mz97*-GAL4, respectively. *ey*^3.5^-GAL4 drives the expression of UAS-*RNAi* transgene in the epithelial monolayer of the eye disc, but not in sub-retinal glia (Fig. 1A and A”). Whereas *Mz*97-GAL4 turns on the expression of UAS-RNAi transgene specifically in WG, and also in some other glial cell types in the optic lobe (Fig. 1B and B”) [12]. To visualize R cells and WG, third-instar eye-brain complexes from knockdown animals were double-stained with MAb 24B10 and anti-Bdl antibodies (Fig. 2A-A”). MAb 24B10 recognizes the R-cell-specific cell adhesion molecule Chaoptin [13], whereas Bdl is specifically expressed in WG at the third-instar larval stage [10]. From the initial screen, we identified thirteen transgenic RNAi lines that disrupted R-cell and/or WG development (Table 1). Three phenotypic classes were observed, including defects in R cells only, in WG only, or in both R cells and WG (see below).

**Table 1:** Identification of *RNAi* lines that disrupted R-cell and/or WG development. The phenotypes were classified into three classes, including defects in R cells only, in WG only or in both R cells and WG

Phenotypic classes	RNAi lines	Genes	Penetrance
R cells only	BDSC# 27503	*N-Cadherin* (*CadN*)	22/22
WG only	BDSC# 28624	*astrocytic leucine-rich repeat molecule (alrm)*	6/17
	BDSC# 34661	*Thrombospondin (Tsp)*	3/8
Defects in both R cells and WG	BDSC# 33416	*amalgam (ama)*	22/22
	BDSC# 60067	*beat-Vc*	9/9
	BDSC# 32860	*domeless (dome)*	11/11
	BDSC# 25781	*Epidermal growth factor receptor (EGFR)*	14/14
	BDSC# 66958	*kuzbanian (kuz)*	7/10
	BDSC# 34965	*Leukocyte-antigen-related-like (LAR)*	24/24
	BDSC# 33642	*myospheroid (mys)*	9/11
	BDSC# 37496	*neuroglian (nrg)*	11/20
	BDSC# 38207	*shotgun (shg)*	7/10
	BDSC# 66968	*SP2353*	3/10

### Knockdown only disrupted R-cell development

From the screen, we identified one RNAi line (i.e. BDSC#27503) whose expression affected the projections of R-cell axons without causing obvious defects in WG development (Table 1, Fig. 2B-B”). In wild type, R-cell axons project through the optic stalk into the developing optic lobe. R1-R6 axons stop in the superficial lamina layer, where their expanded growth cones form a dense and continuous terminal layer. R7 and R8 axons pass through the lamina and elaborate a smooth array of expanded terminals within the deeper medulla layer (Fig. 2A and A”). In knockdown animals targeting *N-Cadherin* (*CadN*) (Fig. 2B and B”), although R-cell axons projected normally through the optic stalk into the developing optic lobe, the organization of R-cell axonal terminals was disrupted. Gaps were frequently observed at the R1-R6 terminal layer. Within the medulla, R7 and R8 axonal terminals failed to form an organized topographic pattern, and their terminal morphology was abnormal (Fig. 2B and B”). This *CadN* knockdown phenotype was identical to that observed in *CadN* loss-of-function mutants reported in previous studies [14].

Although *CadN* knockdown severely disrupted the termination pattern of R-cell axons (Fig. 2B and B”), no obvious defect in WG development was observed in knockdown animals (Fig. 2B’ and B”). In *CadN* knockdown animals, like that in wild type, differentiating WG processes followed R-cell axons from the eye disc into the lamina (Fig. 2B’ and B”). The number of WG processes also appeared normal (Fig. 2B’ and B”).

### Knockdown only disrupted WG development

**Fig. 3:**
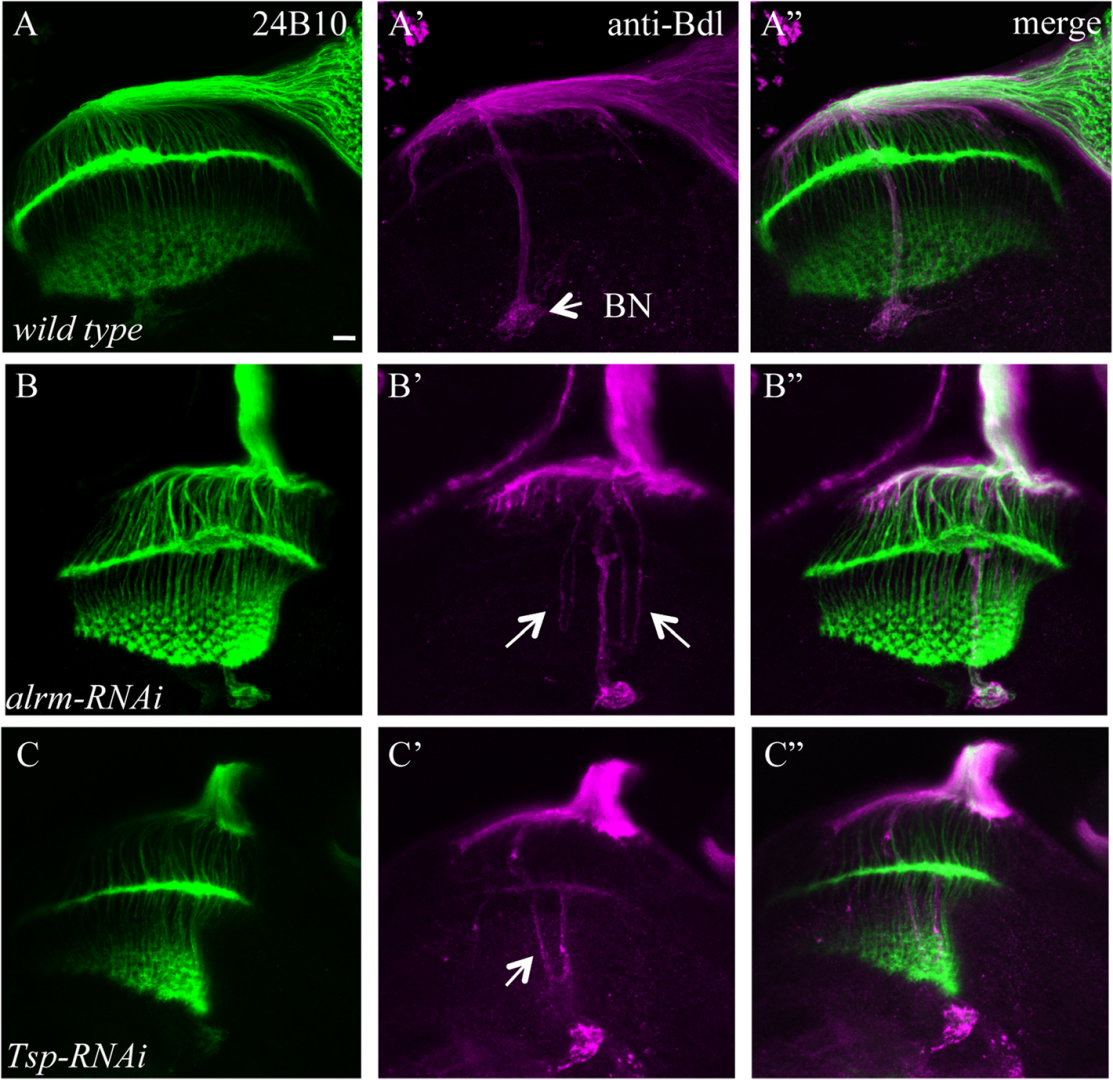
Knockdown affected WG development only. UAS-*RNAi* transgenes were expressed in both R cells and WG under control of *ey*^*3.5*^-GAL4 and *Mz97*-GAL4. 3rd-instar larval eye-brain complexes were double-stained with MAb24B10 (green) and anti-Bdl (magenta). A-A”, wild type. B-B″, an eye-brain complex in which the *RNAi* line (i.e. BDSC# 28624) targeting *alrm* was expressed in both eye disc and WG. In knockdown animals, although R cells in the eye disc developed normally (B and B”), some WG processes failed to cease extension in the lamina, and instead projected aberrantly into the deeper medulla (arrows) (B’). C-C″, an *alrm*-like WG extension phenotype was observed in animals expressing the *RNAi* line (BDSC# 34661) targeting *Tsp*. Scale bar: 20 μm

The expression of BDSC# 28624 or BDSC# 34661 RNAi transgene affected WG projections in the developing optic lobe without disrupting R-cell development (Table 1, Fig. 3B-B”, C-C”). RNAi lines BDSC# 28624 and BDSC# 34661 targeting *astrocytic leucine-rich repeat molecule* (*alrm*) and *Thrombospondin* (*Tsp*), respectively. In wild type (Fig. 3A’ and A”), WG followed R-cell axons from the eye disc through the optic stalk into the developing optic lobe, where they ceased extension at the proximal region of the lamina. In *alrm* (Fig. 3B’ and B”) or *Tsp* knockdown animals (Fig. 3C’ and C”), however, some WG processes extended further into the deeper medulla layer.

### Knockdown disrupted both R cell and WG development

**Fig. 4:**
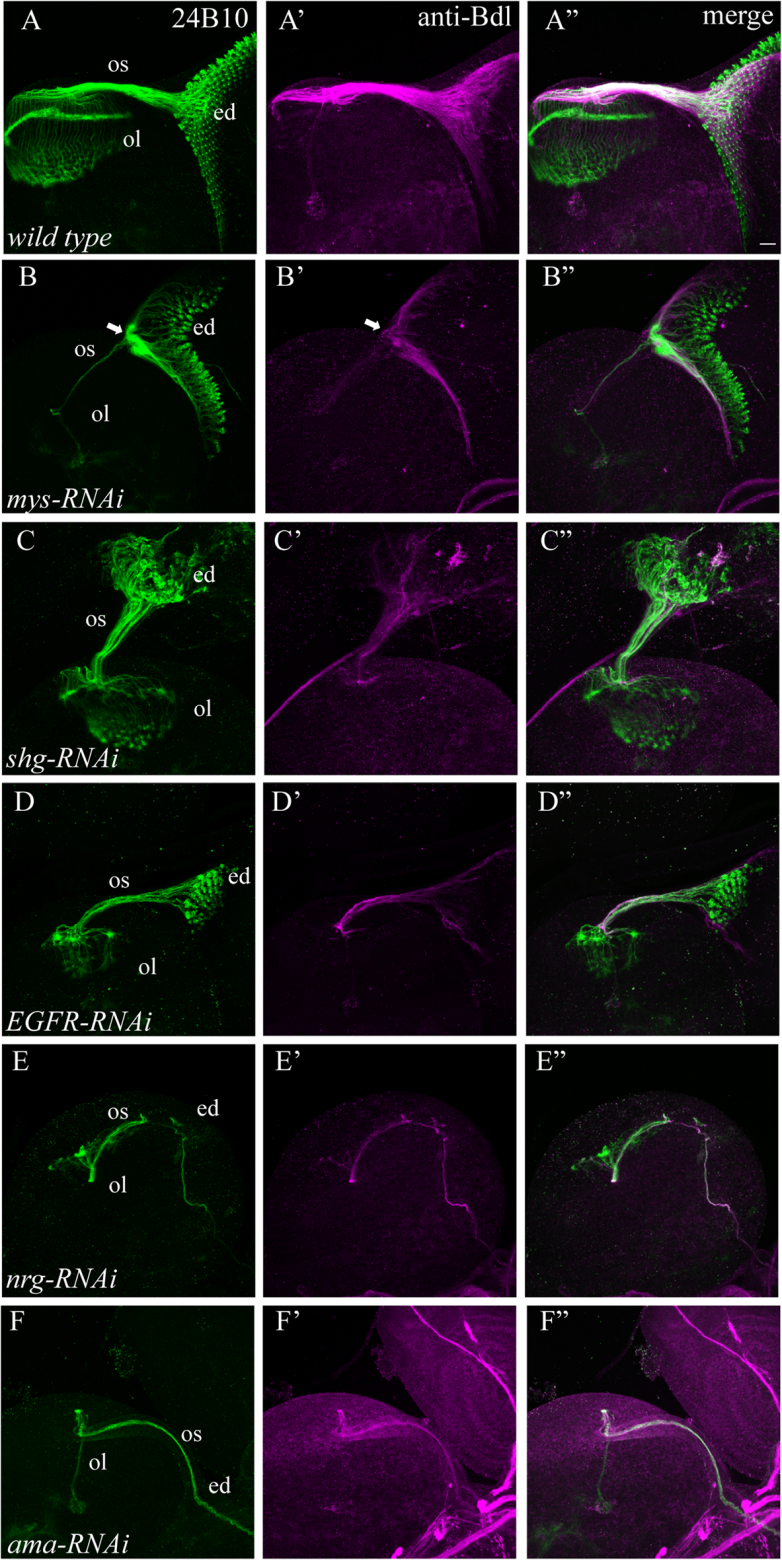
Knockdown affected both R-cell and WG development. UAS-*RNAi* transgenes were driven by both *ey*^*3.5*^-GAL4 and *Mz97*-GAL4. 3rd-instar larval eye-brain complexes were double-stained with MAb24B10 (green) and anti-Bdl (magenta). A-A”, wild type. B-B″, an eye-brain complex in which the *RNAi* line (i.e. BDSC# 33642) targeting *mys* was expressed in the eye disc and WG. While the differentiation of R cells and WG appeared normal, the projection pattern of R-cell axons and WG processes was severely disrupted. Both R-cell axons and WG processes stalled at the posterior end of the eye disc (arrow in B and B’) and failed to enter the optic stalk. C-C″, the organization of R cells and WG was disrupted in animals in which the *RNAi* line (i.e. BDSC# 38207) targeting *shg* was expressed in the eye disc and WG. D-D”, an eye-brain complex in which the *RNAi* line (i.e. BDSC# 25781) targeting *EGFR* was expressed in the eye disc and WG. *EGFR* knockdown decreased the number of differentiating R cells and WG. E-E”, expression of the *RNAi* line (i.e. BDSC# 37496) targeting *nrg* also disrupted the differentiation of R cells and WG. F-F″, expression of the *RNAi* line (i.e. BDSC# 33416) targeting *ama* completely blocked the differentiation of R cells and WG. Abbreviations: ed., eye disc; ol, optic lobe; os, optic stalk. Scale bar: 20 μm

From the screen, we identified ten RNAi lines that disrupted the development of both R cells and WG (Table 1, Fig. 4). These RNAi lines target the genes including *ama*, *beat-Vc, dome*, *EGFR, Leukocyte-antigen-related-like (LAR)*, *kuz*, *myospheroid (mys), nrg, shg,* and *SP2353*.

Among the above ten RNAi lines, the transgene targeting *mys* (i.e. BDSC#33642) did not affect the proliferation and differentiation of R cells and WG (Table 1, Fig. 4B-B”). However, R-cell axons and WG processes stalled at the posterior end of the eye disc and failed to enter the optic stalk. This phenotype is consistent with previous reports that *mys* is required for glial and R-cell axonal migration [15, 16].

In knockdown animals in which the *shg* gene was targeted with *RNAi* transgene (i.e. BDSC# 38207), the organization of R-cell cluster in the eye disc was severely disrupted (Table 1, Fig. 4C and C”). WG processes within the sub-retinal region were disorganized, which frequently formed abnormal large bundles (Fig. 4C’ and C”). R-cell axonal bundles abnormally split within the optic stalk, and the termination pattern of R-cell axons in the optic lobe was also disrupted (Fig. 4C and C”). Compared to that in wild type (Fig. 4A’ and A”), much less WG processes projected through the optic stalk into the lamina (Fig. 4C’ and C”).

The remaining eight RNAi lines caused significant decreases in the number of R cells and WG (Table 1). The severity of WG phenotypes appeared to correlate with that of R-cell phenotypes. Partial loss of developing R cells in *dome, EGFR* (Fig. 4D-D”)*, kuz, or nrg* (Fig. 4E-E”) knockdown animals associated with a decrease in the number of WG processes and the disorganization of WG projections (Table 1). When R cells failed entirely to develop in the eye disc in *ama* (Fig. 4F-F”)*, beat-Vc, LAR* or *SP2353* knockdown animals, complete disappearance of WG in the sub-retinal region was observed (Table 1).

### Testing positive genes with additional RNAi lines

**Fig. 5:**
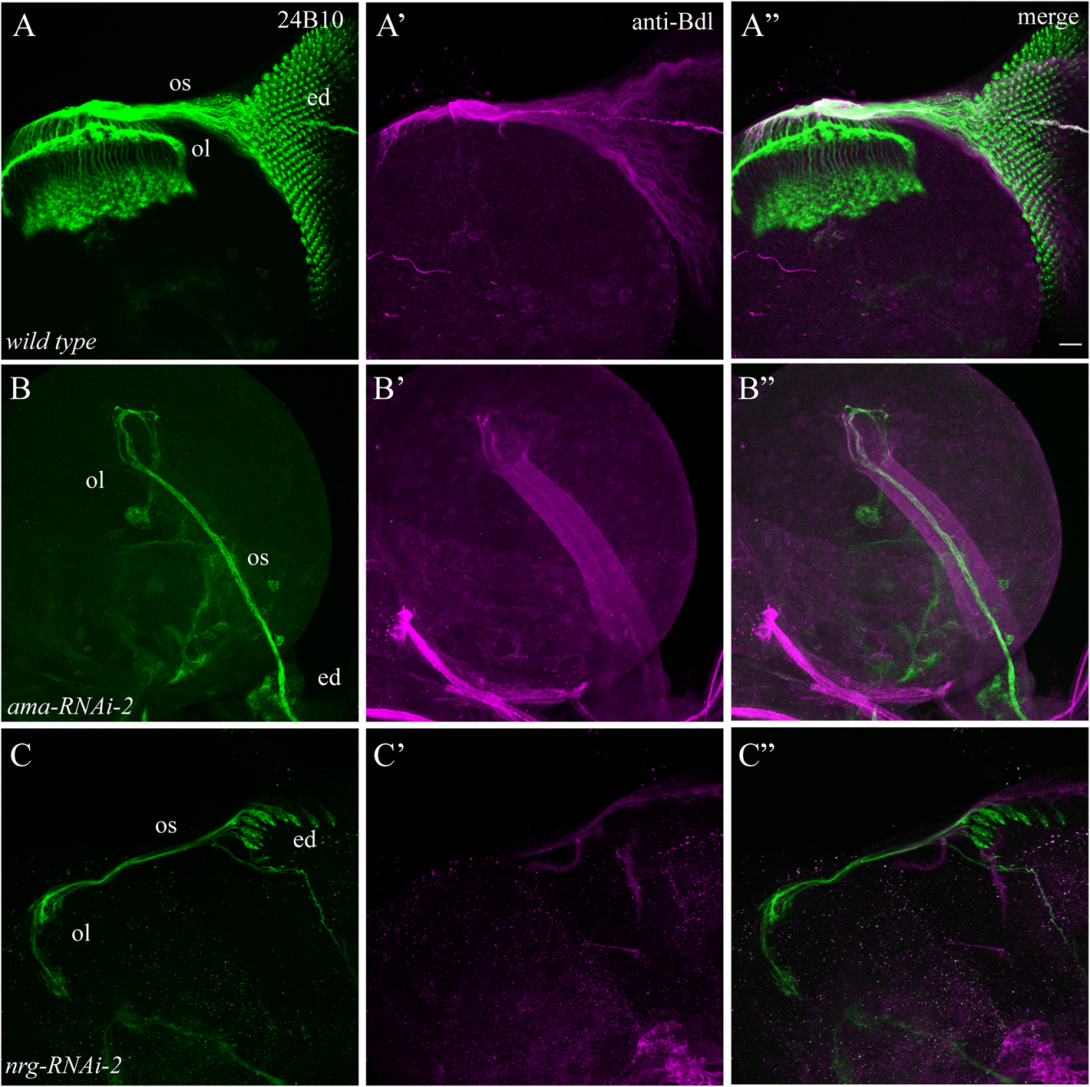
Genes confirmed by additional RNAi lines. UAS-*RNAi* transgenes were under control of both *ey*^*3.5*^-GAL4 and *Mz97*-GAL4. 3rd-instar larval eye-brain complexes were double-stained with MAb24B10 (green) and anti-Bdl (magenta). A-A”, wild type. B-B″, knocking down *ama* using another independent *RNAi* line (i.e. VDRC# 22944 or *ama-RNAi-2*) also completely blocked the differentiation of R cells and WG. C-C″, similar defects in the development of R cells and WG were observed when *nrg* was knocked down by another independent *RNAi* line (i.e. BDSC# 38215 or *nrg-RNAi-2*). Scale bar: 20 μm

To address the possibility that the observed knockdown phenotypes were due to off-target effects, we tested the above genes with additional independent RNAi lines. Only genes whose knockdown with two independent RNAi lines caused similar phenotypes were subject to further analysis. From such analysis, we confirmed seven genes including *ama* (Fig. 5B-B”)*, CadN, dome*, *EGFR, kuz*, *nrg* (Fig. 5C-C”) and *shg* (Table 2).

**Table 2:** Genes confirmed by testing additional *RNAi* lines. To address the possibility of off-target effects, additional *RNAi* lines were tested to examine if knocking down the same gene using at least two independent *RNAi* lines caused similar phenotypes

	RNAi lines	Penetrance
*ama*	BDSC# 33416	22/22
	VDRC# 22944	3/7
*CadN*	BDSC# 27503	22/22
	BDSC# 41982	3/3
*dome*	BDSC# 32860	11/11
	BDSC# 34618	8/8
	BDSC# 31474	2/10
*EGFR*	BDSC# 25781	14/14
	BDSC# 31183	7/9
*kuz*	BDSC# 66958	7/10
	VDRC# 107036	7/9
*nrg*	BDSC# 37496	11/20
	BDSC# 38215	2/11
	BDSC# 28724	1/10
*shg*	BDSC# 38207	7/10
	BDSC# 27689	3/9

### Genes required in the developing eye for the development of R cells and WG

**Fig. 6:**
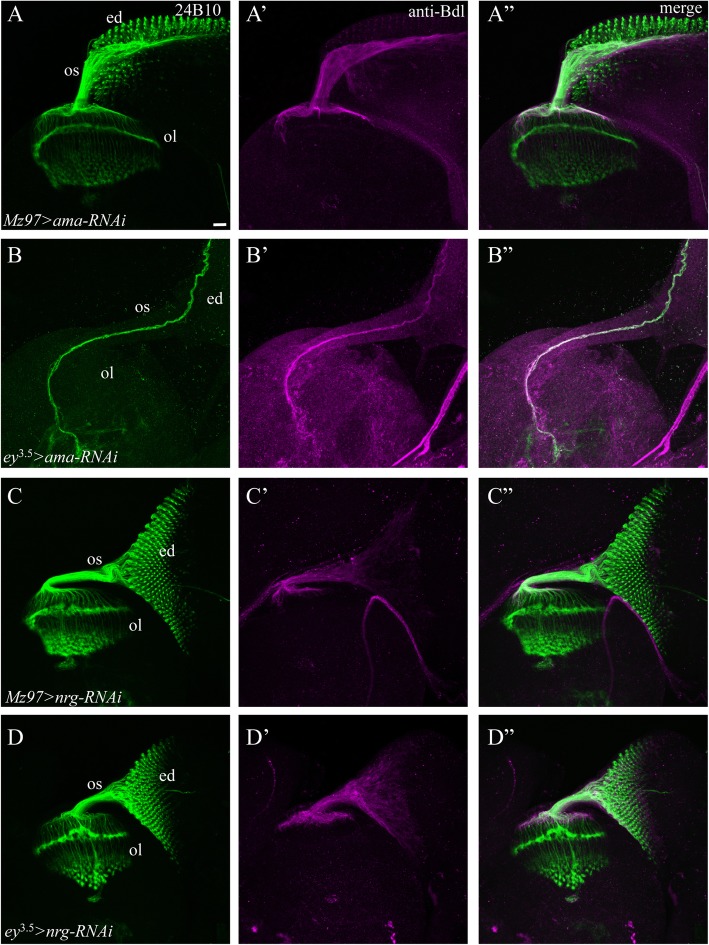
Knockdown in the eye disc only or in WG only. For eye-specific knockdown, UAS-*RNAi* transgenes were under control of *ey*^*3.5*^-GAL4. For WG-specific knockdown, UAS-*RNAi* transgenes were under control of *Mz97*-GAL4. 3rd-instar larval eye-brain complexes were double-stained with MAb24B10 (green) and anti-Bdl (magenta). A-A”, both R cells and WG developed normally when *ama* was only knocked down in WG. B-B″, knocking down *ama* in the eye disc only caused a phenotype identical to that in animals when *ama* was knocked down in both eye disc and WG. C-C″, unlike that in animals in which *nrg* was knocked down in both eye disc and WG (Fig. 4E-E”), the development of R cells and WG appeared normal in animals in which *nrg* was only knocked down in WG. D-D”, the development of R cells and WG occurred normally in animals in which *nrg* was only knocked down in the eye disc. Scale bar: 20 μm

Since all the phenotypes mentioned above were observed when a gene was simultaneously knocked down in the developing eye disc and sub-retinal WG, the results may reflect a role for the gene in the eye disc, WG or both. To distinguish among these possibilities, we performed knockdown experiments by expressing UAS-*RNAi* transgene under control of the eye-specific driver *ey*^3.5^-GAL4 only or the WG-specific driver *Mz97*-GAL4 only. Among the above seven genes, we found that no defect was observed when *ama*, *CadN, dome*, *shg*, *kuz* or *EGFR* was specifically knocked down in WG only (Table 3, e.g. Fig. 6A-A”). When *ama*, *CadN, dome*, *shg*, *kuz* or *EGFR* was specifically knocked down in the eye disc, the phenotypes (Table 3, e.g. Fig. 6B-B”) were very similar to that in animals with gene knockdown in both eye disc and WG.

**Table 3:** Cell-type-specific requirements. UAS-*RNAi* transgenes were expressed under control of the eye-specific driver *ey*^3.5^-GAL4 or the WG-specific driver *Mz97*-GAL4. The penetrance of eye-specific or WG-specific knockdown phenotypes was then compared to that in both eye disc and WG knockdown animals in which UAS-*RNAi* was expressed under control of *ey*^3.5^-GAL4 and *Mz97*-GAL4

Genes	RNAi line	Eye and WG	Eye only	WG only
*ama*	BDSC# 33416	22/22	9/9	0/9
*CadN*	BDSC# 27503	22/22	8/8	0/8
*dome*	BDSC# 32860	11/11	10/10	0/8
*EGFR*	BDSC# 25781	14/14	7/11	0/10
*kuz*	BDSC# 66958	7/10	8/8	0/9
*shg*	BDSC# 38207	7/10	5/10	0/8
*nrg*	BDSC# 37496	11/20	0/19	0/20

These results indicate that *CadN, ama*, *dome*, *shg*, *kuz* and *EGFR* function only in the developing eye disc for the control of R-cell and WG development.

### Genes acted in both developing eye disc and WG for the development of R cells and WG

We also examined cell-type-specific requirements of *nrg*. Interestingly, we found that although knocking down *nrg* simultaneously in the eye disc and WG caused severe defects in R-cell and WG development (Figs. 4 and 5), knocking down *nrg* in WG only (Fig. 6C-C”) or in the eye disc only (Fig. 6D-D”) did not affect the development of R cells nor WG. These results indicate that Nrg functions in both eye and WG for coordinating the development of R cells and WG.

### Knocking down a*ma* or *nrg* disrupted the migration of glia from the optic stalk into the eye disc

To further understand the cause of severe WG phenotype in some knockdown animals, we analyzed the effects of *ama* and *nrg* knockdown on the migration of glia in the developing visual system. Sub-retinal glial cells originate from the optic stalk. At the third-instar larval stage, perineurial glial cells (PG) proliferate in the optic stalk and subsequently migrate from the optic stalk into the sub-retinal space of the eye disc, where they differentiate into WG [8].

**Fig. 7:**
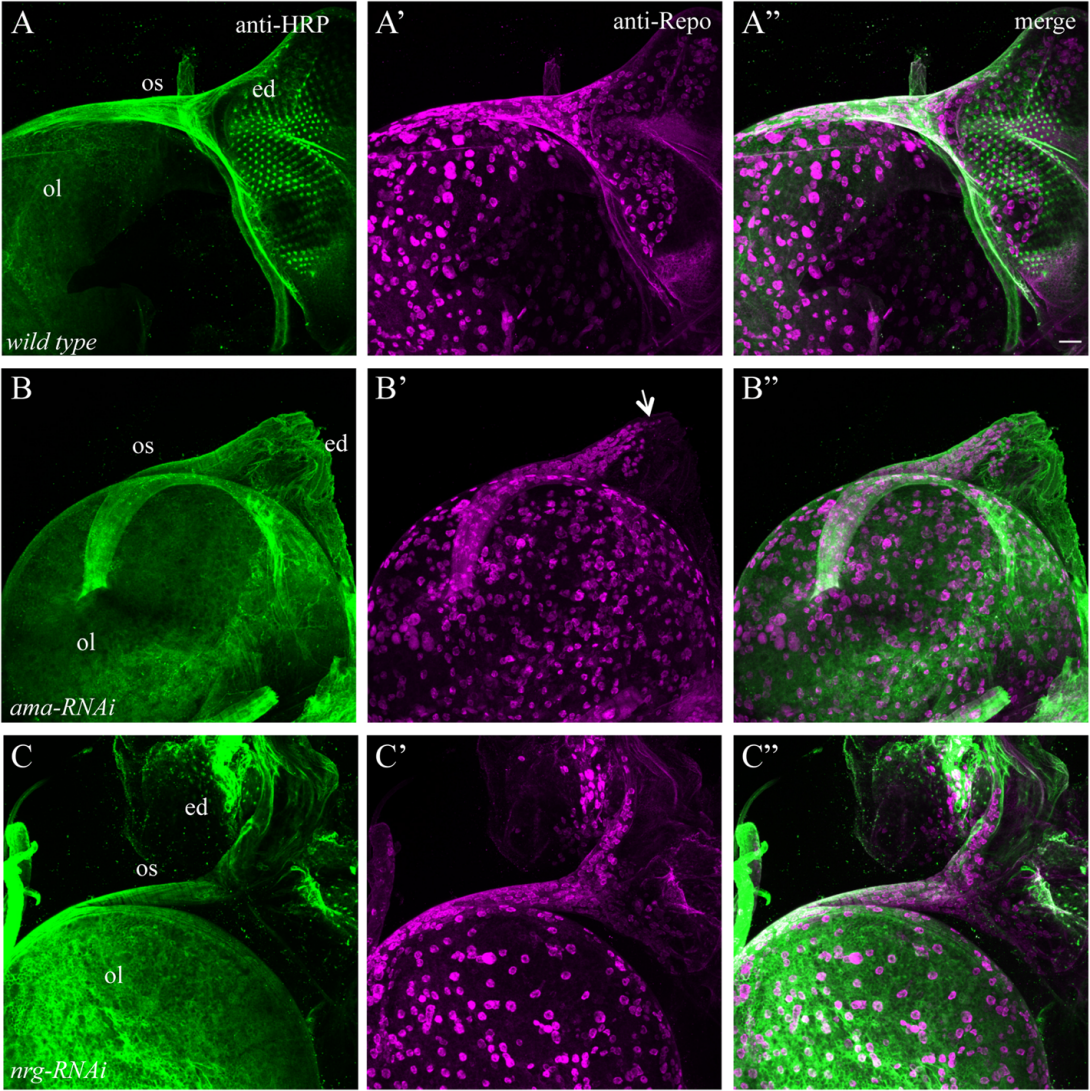
Knocking down *ama* or *nrg* disrupted the migration of glia from the optic stalk into the eye disc. UAS-*RNAi* transgenes were expressed in both R cells and WG under control of *ey*^*3.5*^-GAL4 and *Mz97*-GAL4. 3rd-instar larval eye-brain complexes were double-stained with anti-HRP (green) and anti-Repo (magenta). Anti-HRP and anti-Repo label neuronal processes and all glial cells, respectively. A-A”, in wild-type animals (100%, *n* = 13), glial cells in the optic stalk migrate into the sub-retinal region of the eye disc, where they differentiate into WG after contacting nascent R-cell axons. B-B″, an eye-brain complex in which *ama* was knocked down. The size of the *ama* knockdown eye disc was much smaller than that in wild type. Glial cells accumulated in the optic stalk (arrow) and failed entirely to enter the eye disc (100%, *n* = 9 animals). C-C″, an eye-brain complex in which *nrg* was knocked down in both eye disc and WG. Although the size of the eye disc was similar to that in wild type, the number of glial cells in the sub-retinal region was significantly decreased in *nrg* knockdown animals (9 out 12 animals examined). Scale bar: 20 μm

The distribution of glial cells in the third-instar eye-brain complexes was visualized using anti-Repo antibody, which recognizes the nuclear protein Repo expressed in all glial cells. We found that glial cells accumulated abnormally at the entry point of the eye disc in *ama* knockdown animals (Fig. 7B’ and B”). The size of the eye disc in *ama* knockdown animals (Fig. 7B-B”) was much smaller than that in wild type (Fig. 7A-A”), and no R cell was present in the *ama* knockdown eye disc (Fig. 7B). We also examined Repo staining pattern in animals in which *nrg* was knocked down in both eye disc and WG. Although the size of the eye disc appeared relatively normal in *nrg* knockdown animals, the number of Repo-positive cells in the eye disc was significantly decreased (Fig. 7C’ and C”). These results suggest that the failure of glial migration may contribute at least in part to the WG phenotypes observed in some knockdown animals.

## Discussion

In this study, we performed a transgenic RNAi screen to search for novel cell-surface players that coordinate the development of neurons and glia in the developing *Drosophila* visual system. Previous systematic genetic screens utilizing the fly eye as a model led to the identification of many key components of evolutionarily conserved pathways in regulating neuronal development (e.g. [17, 18]). However, none of the previous screens had systematically examined if genes regulating neuronal development in the eye are also required for the development of sub-retinal WG. And no systematic genetic screen has ever been performed to identify WG-derived factors that modulate neuronal development in the fly eye. By screening a collection of RNAi lines targeting 177 genes encoding for secreted factors and cell-surface receptors, we identified 13 knockdown lines that caused defects in the development of R cells, WG or both. Subsequent analysis of additional RNAi lines confirmed seven genes, including *ama, CadN, dome*, *EGFR, kuz*, *nrg* and *shg*. Among them, *nrg* functions in both eye disc and WG to coordinate the development of R cells in the eye disc and WG in the sub-retinal region. The remaining six genes are only required in the eye disc for regulating R-cell and/or WG development.

*CadN* and *shg* encode for the fly orthologs of mammalian N-Cadherin and E-Cadherin, respectively, both of which belong to the classical Cadherin family of cell adhesion molecules*.* Consistent with previous loss-of-function studies [14], we show that. knocking down *CadN* disrupted the organization of R-cell axonal terminals in the optic lobe, and also caused abnormal axonal terminal morphology. However, the fact that WG projected normally in *CadN* knockdown animals suggests that unlike that of Tutl and Bdl [9, 10], the adhesive property of CadN is not involved in mediating the recognition between R-cell axons and WG for the extension of WG processes.

Unlike *CadN* knockdown that only affected R-cell axonal projection pattern in the optic lobe, *shg* knockdown severely disrupted the organization of R cells in the developing eye disc. Like its mammalian homolog E-Cadherin, Shg is well known for its role in regulating cell-cell adhesion during epithelial morphogenesis [19]. For instance, Shg is required for the establishment of adherens-junction (e.g. [20]). Thus, one likely cause of eye phenotype is the failure of Shg-mediated adherens-junction formation that is required for the organization of R-cell clusters. In addition to R-cell developmental defects in the eye, the extension of WG processes also appeared to be affected in *shg* knockdown animals. There are two possible explanations for the WG phenotype. First, since WG processes follow R-cell axons along the path of migration, the disorganization of R-cell axonal bundles in *shg* knockdown eye disc may present physical barriers for WG extension. And second, Shg, like Tutl, may mediate specific recognition between R-cell axons and WG, thus facilitate the migration of WG processes along R-cell axons.

Our results indicate that *ama, dome*, *EGFR* and *kuz* function in the developing eye disc, but not in WG, for the proper development of R cells and WG. The severity of defects in WG development appeared to correlate with the reduction in the number of differentiating R cells in the eye disc. The R-cell developmental defects suggest a role for these genes in promoting cell proliferation and differentiation in the eye disc. Indeed, *dome*, *EGFR* and *kuz* have been shown previously to function in key signaling pathways for regulating cell growth and differentiation [21]. For instance, *dome* encodes for a transmembrane receptor for the cytokine-like ligand Upd, and functions in activating the intracellular JAK/STAT pathway [22]. Upd/Jak/STAT signaling has been shown to be required for promoting morphogenetic furrow initiation in the eye disc [23]. The EGFR receptor tyrosine kinase is a well-known regulator that promotes R-cell proliferation and differentiation in the developing eye disc [24]. And *kuzbanian (kuz)* encodes for a member of membrane-anchored metalloprotease-disintegrins (ADAMs) [25]. It is reported that *kuz* is required for Notch activation [26]. Previous studies show that *kuz* mutation severely disrupted the regular array of ommatidia in the adult eye [25, 27]. Unlike *dome*, *EGFR* and *kuz, ama* has not been previously implicated in promoting cell proliferation and differentiation. *ama* encodes for a secreted Ig-like protein [28] and is reported to play a role in regulating axonal fasciculation and pathfinding [29, 30]. Our results suggest that *ama* may also function as a growth factor to promote eye morphogenesis.

One likely explanation for the correlation between WG defects and the decrease in the number of R cells in the eye disc is the requirements of R-cell-derived signals for WG differentiation and projection. It is shown that R-cell-derived FGF8-like ligand Thisbe is required for the differentiation of WG [8]. And Tutl protein expressed on R-cell axons binds to the WG-specific Bdl in promoting WG extension [9]. Thus, a decrease in the number of R cells may reduce the levels of R-cell-derived factors for WG differentiation and extension. Additionally, the glial migration defects observed in *ama* and *nrg* knockdown animals, suggest that a failure of glial migration due to defective eye development may also contribute to the defects in WG development.

The results from Cell-type-specific knockdown suggest that Nrg may mediate specific interactions between WG and certain cell types (R cells and/or other cell types) in the eye disc for coordinating the development of R cells and WG. *nrg* encodes for the fly ortholog of the mammalian neural cell adhesion molecule L1, which is a member of the Ig superfamily [31]. In *Drosophila*, it has been shown that Nrg is involved in regulating axonal pathfinding and synaptic development (e.g. [32–34]). Since Nrg/L1-family cell adhesion molecules possess both homophilic and heterophilic binding activity [35, 36], Nrg may mediate the interactions between WG and R cells (or other cell types in the eye disc) via Nrg-Nrg homophilic binding, or via heterophilic binding between Nrg and another cell-surface receptor. Interestingly, no phenotype was observed when *nrg* was knocked down in the eye only or in WG only. One possible explanation is that when Nrg on one cell type (e.g. eye-disc cell) was removed, opposing cell type (e.g. WG) still has Nrg on its surface, which allows Nrg to mediate eye-WG interactions by binding to another cell-surface receptor on opposing cell surface. The Nrg-mediated cell-cell recognition between WG and eye-disc cells may directly promote cell proliferation and differentiation for coordinating the development of R cells and WG. Alternatively, Nrg may function indirectly by antagonizing the effects of pro-apoptotic factors. Future studies will be needed to distinguish between these possibilities.

In conclusion, our RNAi screen targeting secreted proteins and cell-surface receptors identified specific phenotypes in the development of R cell, WG or both. This study presents an excellent starting point for further molecular and genetic dissection of the action of the identified genes and sheds new light on the general mechanisms underlying the coordinated development of neurons and glia in vertebrates and invertebrates.

## Materials and methods

### Genetics

All the fly stocks are maintained at 25 °C with 50% humidity and 12/12 h light-dark cycle. The RNAi stocks were obtained from Bloomington Drosophila Stock Center (BDSC) and Vienna Drosophila Resource Center (VDRC) (Additional file 1: Table S1). GAL4 lines (*Mz97*-GAL4; *ey*^3.5^-GAL4/*Tb, ey*^3.5^-GAL4/*Tb*, *Mz97*-GAL4; +/+) were either obtained from BDSC or generated in our previous studies [9, 10].

### Histology

Whole-mount eye-brain complexes from third-instar larvae were dissected and stained as described previously [9].

### Immunostaining

Antibodies were used at following dilutions: mouse MAb24B10 (1:100; Developmental Studies Hybridoma Bank or DSHB Cat#24B10), rabbit polyclonal anti-GFP (1:750; Molecular Probes, Cat#A-11122), rabbit polyclonal anti-Bdl (1:1000), Alexa 647-conjugated goat anti-HRP (1:500; Jackson ImmunoResearch Cat#123–605-021). Secondary antibodies including Alexa Fluor 488 goat anti-Mouse IgG (Invitrogen, Cat# A11001) and Alexa Fluor 647 goat anti-Rabbit IgG (Invitrogen, Cat# A20991), were used at 1:500 dilution.

### Confocal microscopy

Epifluorescent images were analyzed by confocal microscopy (Olympus FV1000). Photo stacks are performed using Z-stack projection by FluoView software.

## Supplementary information

**Additional file 1: Table S1.** A list of transgenic RNAi lines targeting 177 genes encoding for secreted proteins and cell-surface receptors. The positive genes are highlighted.

## Data Availability

The datasets supporting the conclusion of this study are included in this article.

## Abbreviations

alrm: Astrocytic leucine-rich repeat molecule
ama: Amalgam
Bdl: Borderless
BN: Bolwig’s Nerve
CadN: N-Cadherin
dome: Domeless
ed.: Eye disc
EGFR: Epidermal growth factor receptor
kuz: Kuzbanian
la: Lamina
me: Medulla
mys: Myospheroid
nrg: Neuroglian
ol: Optic lobe
os: Optic stalk
PG: Perineurial glia
R cells: Photoreceptors
shg: Shotgun
Tsp: Thrombospondin
Tutl: Turtle
WG: Wrapping glia
