# Supplementary material for: An RNAi screen for secreted factors and cell-surface players in coordinating neuron and glia development in Drosophila
Source: Mol Brain. 2020 Jan 3;13:1. doi: 10.1186/s13041-019-0541-5 (PMC6942347; doi:10.1186/s13041-019-0541-5)
Supplement: Supplementary file 1 — Additional file 1: Table S1. A list of transgenic RNAi lines targeting 177 genes encoding for secreted proteins and cell-surface receptors. The positive genes are highlighted. [file 13041_2019_541_MOESM1_ESM.docx]

Additional file 1: Table S1. A list of transgenic RNAi lines targeting 177 genes encoding for secreted proteins and cell-surface receptors. The positive genes are highlighted.
